# Surgery Quality and Tumor Status Impact on Survival and Local Control of Resectable Liposarcomas of Extremities or the Trunk Wall

**DOI:** 10.1007/s11999-012-2592-0

**Published:** 2012-09-13

**Authors:** Piotr Rutkowski, Sławomir Trepka, Konrad Ptaszynski, Milena Kołodziejczyk

**Affiliations:** 1 Department of Soft Tissue/Bone Sarcoma and Melanoma, Maria Sklodowska-Curie Memorial Cancer Center and Institute of Oncology, Roentgena 5, 02-781 Warsaw, Poland; 2 HolyCross Oncological Center, Department of Surgical Oncology, Kielce, Poland; 3 Department of Pathology, Maria Sklodowska-Curie Memorial Cancer Center and Institute of Oncology, Warsaw, Poland; 4 Department of Radiotherapy, Maria Sklodowska-Curie Memorial Cancer Center and Institute of Oncology, Warsaw, Poland

## Abstract

**Background:**

The 5-year survival rates for localized liposarcomas reportedly vary from 75% to 91% with histologic grade as the most important prognostic factor. However, it is unclear which other factors, including the initial surgery quality and recurrent tumors, influence survival in localized liposarcomas (LPS).

**Questions/Purposes:**

We analyzed factors (including AJCC staging system) influencing survival and local control of resectable LPS of the extremities/trunk wall and the impact of surgery quality and tumor status and type of disease recurrences according to pathological subtype.

**Methods:**

We retrospectively reviewed 181 patients with localized LPS: 110 were treated for primary tumors, 50 for recurrent tumors, and 21 for wide scar resection after unplanned nonradical resection. We determined survival rates and examined factors influencing survival. The minimum followup was 4 months (median, 52 months; range, 4–168 months).

**Results:**

Five-year disease-specific (DSS), disease-free (DFS), and local relapse-free survival (LRFS) rates were: 80%, 58%, and 75%, respectively. Five-year local relapse-free survival rates for primary versus clinically recurrent tumor versus scar after nonradical resection were: 86.1%, 52.1%, and 73.3%, respectively. The following were independent negative prognostic factors for DSS (AJCC Stage ≥ IIb), DFS (Grade 3; clinical recurrence; skin infiltration), and LRFS (clinical recurrence; R1 resection). An unplanned excision, although influencing local relapse-free survival, had no impact on disease-specific survival (calculated from date of first excision 5-year rate of 80%, considering impact of combined treatment of clinical recurrence/scar).

**Conclusions:**

We confirmed the value of AJCC staging for predicting disease-specific survival in extremity/trunk wall LPS. Radical reresection of scar after nonradical primary tumor resection (+ radiotherapy) seems to improve disease-free and local relapse-free survival in liposarcomas. Patients with unplanned excision can be cured when referred to a sarcoma unit.

**Level of Evidence:**

Level IV, prognostic study. See the Guidelines for Authors for a complete description of levels of evidence

## Introduction

Soft tissue sarcomas, a heterogeneous group of malignancies, are challenging to diagnose and treat because of their rarity, various clinical presentations, disease course, and multiple pathological subtypes. Liposarcomas are the most common soft tissues tumors (with the exception of gastrointestinal stromal tumors) [16] and well-defined pathologically type of soft tissue sarcomas occurring in adults. Like with other sarcomas, treatment is recommended in referral centers with multidisciplinary expertise [2]. However, in reality, a substantial number of patients with primary soft tissue sarcomas are still treated in nonreferral centers by unplanned resection of the tumor and then referred to tertiary institutions [20, 28]. Several contemporary studies, each of which included more than 100 cases, analyzed the outcomes of patients with localized liposarcomas [7, 9, 12, 17, 18]. These studies suggest 5-year survival rates ranged from 75% to 91% with histologic grade as the most important prognostic factor.

The American Joint Committee on Cancer (AJCC) staging system recently proposed a new staging system for soft tissue sarcomas incorporating a three-tiered grading system and nodal metastases classified as Stage III disease [4, 5]. This system has been never tested for evaluation of cohort of localized liposarcomas. It is also unclear how other prognostic factors, including the quality of the initial surgery (ie, complete or incomplete excision) and other factors, including recurrent tumors, influence survival in localized soft tissue sarcomas. We have evaluated in the study homogenous series of liposarcomas originating from extremities or the trunk wall, which were analyzed jointly because of similar prognosis and approach, contrary to retroperitoneal or head and neck liposarcomas [17].

The aims of the study were to determine: (1) prognostic factors having an influence on disease-specific survival, disease-free survival, and local relapse-free survival, including the new AJCC staging system, based on a homogenous cohort of localized, resectable liposarcomas of the extremities/trunk wall treated with curative intent in a tertiary referral sarcoma center; (2) the impact of quality of surgery and tumor status (primary versus clinically recurrent versus scar after inadequate surgery); and (3) type of disease recurrences according to pathological subtype of liposarcoma.

## Patients and Methods

We retrospectively reviewed all 190 adult patients with localized, resectable liposarcomas originating from the extremities or trunk wall treated with curative intent in a tertiary sarcoma center between 1996 and 2008. Eight patients were excluded owing to loss to followup after surgery. These exclusions left 181 patients with complete clinical-pathological data (Table 1). Median age at diagnosis of the 181 patients was 58 years (range, 18–89 years). Most primary tumors were located in the lower extremity (76%) followed by the trunk wall (14%) and upper extremity (10%). At presentation in the tertiary center, 110 patients (61%) had untreated primary tumors, 50 (27.5%) had clinical local recurrence after resection at an outside facility, and 21 (11.5%) had only scar (without clinically detectable tumor) after primary nonradical unplanned resection at an outside sarcoma center. All cases were classified according to the AJCC staging system 7^th^ edition [4, 5]. Minimum followup was 4 months (median, 52 months; range, 4–168 months; only 11 survivors, 6% had minimum followup less than 12 months). No patients were recalled specifically for this study; all data were obtained from medical records. Analysis of clinicopathologic data had been approved by the local bioethical committee according to Good Clinical Practice Guidelines.

**Table 1:** Patient characteristics

Factors	Number of patients	Percent
Age (years)		
0–49	69	38
> 49–59	47	26
> 59–89	65	36
Sex		
Female	88	49
Male	93	51
Primary tumor location		
Lower extremity	138	76
Upper extremity	18	10
Trunk	25	14
Histological grade		
1	64	35
2	51	28
3	66	37
Tumor size (cm)		
0–5	26	14
> 5–50	155	86
Tumor status at the beginning of treatment in sarcoma center		
Primary tumor	110	61
Clinical recurrence	50	27.5
Scar after primary nonradical resection	21	11.5
Tumor biopsy before primary surgery		
No	73	40
Yes	108	60
Surgical resection margins		
R0	148	82
R1	33	18
Histological subtype		
Myxoid/round-cell	104	57
Pleomorphic	49	28
Well-differentiated	27	15
Skin infiltration		
No	170	94
Yes	11	6
AJCC stage		
Ia	14	7.5
Ib	50	27.5
IIa	10	5.5
IIb	48	27
III	58	32
Adjuvant radiotherapy		
No	29	16
Yes	152	84
Initial level of hemoglobin		
Decreased	16	9
Normal	165	91

AJCC = American Joint Committee on Cancer.

All 181 patients underwent wide surgical resection (whenever possible) combined, after multidisciplinary assessment, with adjuvant radiotherapy (pre- or postoperative) in the majority of cases (only five extremity amputations were primarily performed). Patients did not receive any adjuvant chemotherapy. We considered wide excision combined with adjuvant external beam radiation therapy as standard treatment in the majority of soft tissue sarcomas as required by national and international guidelines [2, 19, 30] and 154 of the 181 patients had additional radiotherapy (with exception of all patients in Stage Ia and part in Stage Ib) based on the decision of the multidisciplinary team. In cases of positive margins after surgery preceded by preoperative surgery, the additional boost on the tumor bed was added in individual cases.

Followup information was obtained during regular outpatient visits. Routine surveillance was recommended every 3 months during the first 2 years, every 4 months in Year 3, every 6 months in Years 4 to 5, and thereafter annually. Patients with Stage IA liposarcomas were seen every 6 months after the first year. Posttreatment followup consisted of physical examination and routine imaging investigations. We used CT or radiography for chest wall tumors, abdominal cavity evaluation in myxoid/round-cell liposarcoma subtype (ultrasound alternating with CT), and ultrasound or MR for wide scar resection according to national guidelines [30]. All available medical and histopathological records were reviewed; pathological diagnoses were reconfirmed in our center.

**Fig. 1:**
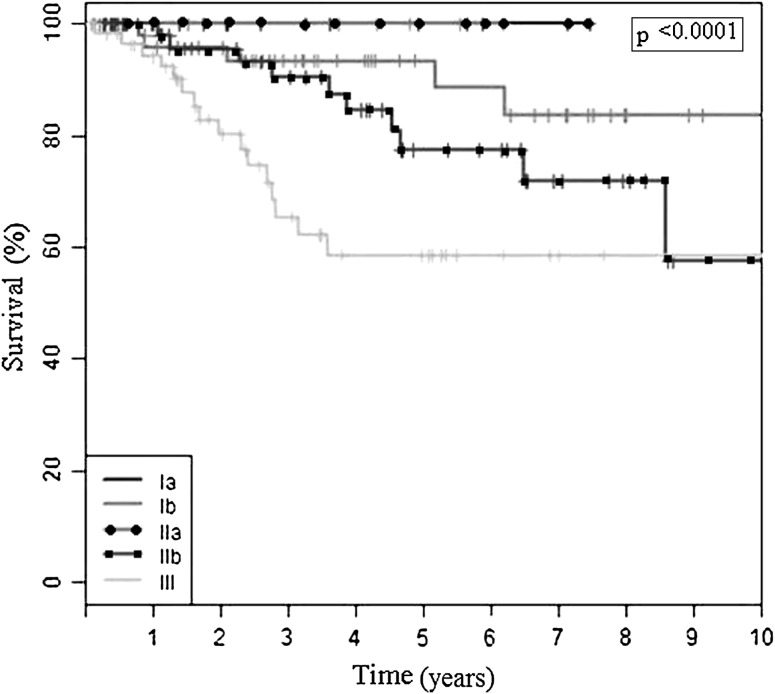
Kaplan-Meier curves show disease-specific survival according to the AJCC staging system: 5-year DSS for Stage Ia and IIa was: 100%, for Ib: 93.4% (95% CI, 86.4%–100.0%), for IIb: 77.5% (95% CI, 64.6%–93.0%), and for III: 58.5% (44.2%–77.3%). DSS = disease-specific survival.

For the survival analysis, Kaplan-Meier estimator was used with the log-rank tests to compare survival between subgroups of patients (listed subsequently). We determined disease-specific survival, disease-free survival, and local recurrence-free survival calculated from the date of tumor resection at the referral center to the date of death resulting from disease, recurrence, or local relapse, respectively, or to the last followup date. We also calculated disease-specific survival from the date of the first tumor resection (outside or in referral center) to the date of death resulting from disease progression or last followup. All deaths from other reasons were recorded as censored. Clinical and pathological parameters as follows: sex, age (< 49 versus 49–59 versus > 59 years), primary tumor site (extremity versus trunk wall; lower versus upper extremity), histological grade (1 versus 2 versus 3), tumor size (≤ 5 versus > 5 cm), tumor status at the beginning of therapy in the tertiary center (primary tumor versus clinical recurrence versus scar after nonradical surgery), the fact of biopsy before primary surgery (yes versus no), margin status at final surgery (R0, microscopically radical resection versus R1, microscopically nonradical, but macroscopically radical resection), histological subtype (myxoid/round cell versus pleomorphic versus well-differentiated), skin infiltration by the tumor/ulceration (no versus yes), AJCC staging groups (Ia versus Ib versus IIa versus IIb versus III), and initial level of hemoglobin in peripheral blood (normal versus decreased) were tested as factors affecting patient survival. For statistical reasons, myxoid and round cell liposarcomas were analyzed together, because the extent of myxoid/round cell component is generally considered a continuum of the same clinical/molecular entity; the percentage of these cells is somewhat arbitrarily recognized, especially on the basis of Tru-cut biopsy [21], because a major proportion of patients had recurrent tumor or had undergone preoperative radiotherapy after biopsy. All 181 patients were included in analysis without any other preliminary selection. We first determined factors influencing disease-specific survival (Table 2) and local recurrence-free survival (Table 3) using univariate analyses. The AJCC Stages IIb and III (p < 0.001) (Fig. 1) and Grade 3 tumors (p < 0.001) were related to the shortest disease-specific survival. In multivariate analysis of the factors associated with survival after resection, we used Cox proportional hazard models applying the stepwise model-building procedure including all covariates significant at the 10% level in univariate analysis. Two-way interactions were then considered in the model. All statistical analyses were performed using the R 2.11 statistical program (R Development Core Team 2010; www.R-project.org).

**Table 2:** Disease-specific survival according to clinical-pathological factors

Factor	5-year survival	95% confidence interval	p value
Age (years)			
0–49	79.7%	69.3%–91.6%	0.81
49–59	80.6%	68.4%–94.8%	
59–89	78.1%	65.7%–92.9%	
Sex			
Female	86.2%	78.0%–95.2%	0.05
Male	71.7%	60.8%–84.6%	
Primary tumor site			
Extremity	78.3%	70.7%–86.8%	0.96
Trunk wall	83.0%	66.5%–100.0%	
Primary tumor site (extremities only)			
Lower extremity	77.2%	69.2%–86.0%	0.19
Upper extremity	100.0%	–	
Histological grade			
1	94.6%	88.9%–100.0%	< 0.0001
2	83.6%	72.0%–97.0%	
3	57.5%	43.8%–75.4%	
Tumor size (cm)			
0–5	100.0%	–	0.04
> 5–50	75.8%	67.5%–85.2%	
Tumor status at beginning of therapy in tertiary center			
Primary tumor	79.4%	70.5%–89.3%	0.04
Clinical recurrence	71.1%	57.7%–87.6%	
Scar after nonradical surgery	100.0%	–	
Biopsy before primary surgery			
No	77.8%	67.3%–90.0%	0.62
Yes	81.0%	72.5%–90.5%	
Radical surgery margins status			
R0	80.5%	72.2%–88.6%	0.58
R1	77.5%	63.0%–95.3%	
Histological subtype			
Myxoid/round cell	78.2%	68.6%–89.1%	0.04
Pleomorphic	67.5%	52.5%–86.8%	
Well-differentiated	95.7%	87.7%–100.0%	
Skin infiltration/ulceration			
No	80.6%	72.4%–87.6%	0.29
Yes	62.3%	35.5%–100.0%	
AJCC staging groups			
Ia	100.0%	–	< 0.0001
Ib	93.4%	86.4%–100.0%	
IIa	100.0%	–	
IIb	77.5%	64.6%–93.0%	
III	58.5%	44.2%–77.3%	
Initial level of hemoglobin			
Decreased	76.6%	55.6%–100.0%	0.85
Normal	79.1%	71.5%–87.4%	

R0 = microscopically radical resection; R1 = microscopically nonradical, but macroscopically radical resection; AJCC = American Joint Committee on Cancer.

**Table 3:** Local recurrence-free survival according to clinical-pathological factors

Factor	5-year survival	95% confidence interval	p value
Age (years)			
0–49	71.7%	60.0%–85.7%	0.18
49–59	84.9%	73.3%–98.3%	
59–89	65.6%	48.7%–88.2%	
Sex			
Female	78.4%	68.4%–89.9%	0.29
Male	68.6%	56.0%–84.2%	
Primary tumor site			
Extremity	75.7%	67.1%–85.5%	0.10
Trunk wall	59.6%	39.7%–89.5%	
Primary tumor site (extremities only)			
Lower extremity	75.3%	66.2%–85.6%	0.59
Upper extremity	85.9%	69.5%–100.0%	
Histological grade			
1	87.6%	78.7%–97.5%	0.04
2	69.0%	54.6%–87.2%	
3	60.1%	44.3%–81.5%	
Tumor size (cm)			
0–5	84.6%	67.1%–100.0%	0.19
5–50	71.3%	61.8%–82.2%	
Tumor status at beginning of therapy in tertiary center			
Primary tumor	86.1%	78.5%–94.5%	0.01
Clinical recurrence	52.1%	36.6%–74.3%	
Scar after nonradical surgery	73.3%	53.6%–100.0%	
Biopsy before primary surgery			
No	58.1%	45.7%–74.0%	< 0.0001
Yes	86.1%	77.0%–96.3%	
Radical surgery margins status			
R0	77.5%	68.7%–87.4%	0.01
R1	60.9%	44.8%–82.6%	
Histological subtype			
Myxoid/round cell	72.5%	60.9%–86.3%	0.03
Pleomorphic	55.0%	38.2%–79.1%	
Well-differentiated	95.2%	86.6%–100.0%	
Skin infiltration/ulceration			
No	76.2%	65.8%–83.8%	0.13
Yes	64.3%	40.9%–100.0%	
AJCC staging groups			
Ia	85.7%	63.3%–100.0%	0.10
Ib	87.9%	78.5%–98.5%	
IIa	80.0%	51.6%–100.0%	
IIb	67.0%	52.1%–86.3%	
III	59.8%	43.3%–82.7%	
Initial level of hemoglobin			
Decreased	76.1%	56.6%–99.7%	0.76
Normal	72.6%	63.5%–82.9%	

R0 = microscopically radical resection; R1 = microscopically nonradical, but macroscopically radical resection; AJCC = American Joint Committee on Cancer.

## Results

**Fig. 2A–C:**
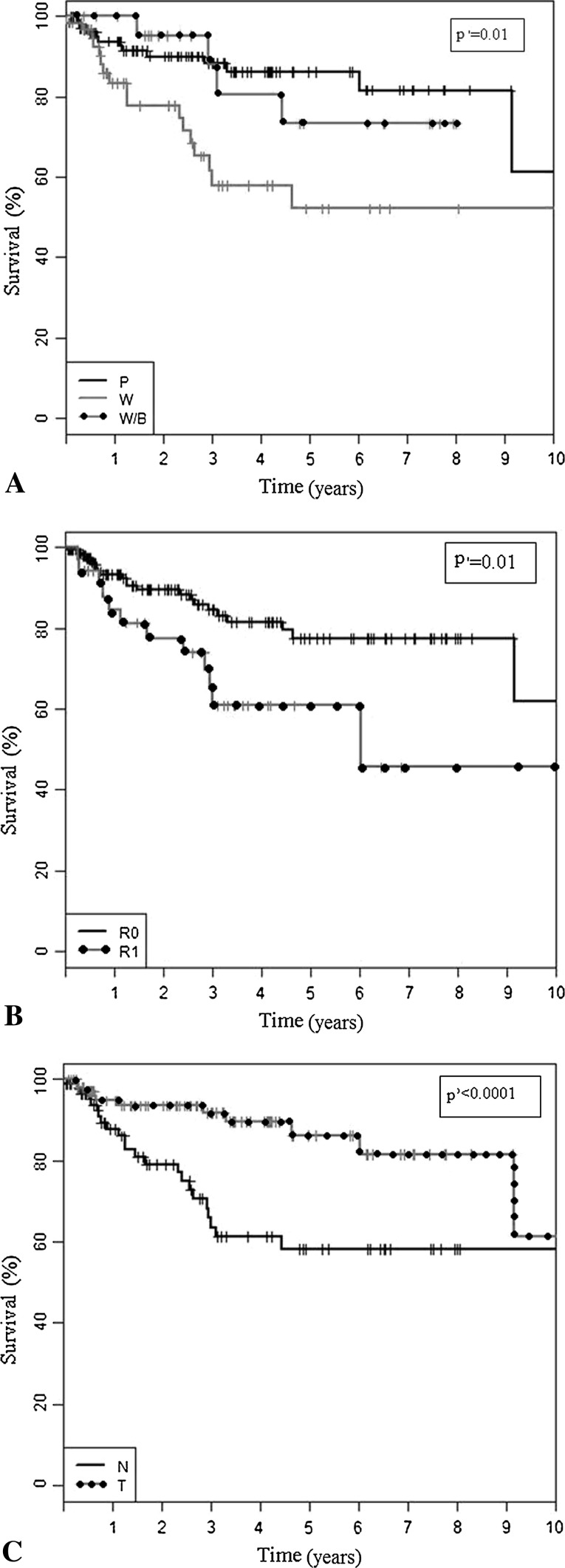
Kaplan-Meier curves show local recurrence-free survival according to: (**A**) tumor status at treatment start in referral center (P = primary tumor; W = clinical recurrence; W/B = scar after nonradical prereferral resection); 5-year LRFS for primary tumor was: 86.1% (95% CI, 78.5%–94.5%), for clinical recurrence: 52.1% (95% CI, 36.6%–74.3%), and for scar after nonradical surgery: 73.3% (95% CI, 53.6%–100.0%); (**B**) surgical margins (R0 = microscopically radical resection; R1 = microscopically nonradical, but macroscopically radical resection); 5-year LRFS for R0 was: 77.5% (95% CI, 68.7%–87.4%), and for R1: 60.9% (95% CI, 44.8%–82.6%); (**C**) preoperative biopsy (unplanned or planned surgery; N = no preoperative biopsy; T = preoperative biopsy performed); 5-year LRFS for performed preoperative biopsy was: 86.1% (95% CI, 77.0%–96.3%%) and for lack of preoperative biopsy: 58.1% (95% CI, 45.7%–74.0%). LRFS = local relapse-free survival.

Estimated 5-year disease-specific survival rate was 80% (95% CI, 72.0%–86.7%), 5-year disease-free survival rate was 58% (95% CI, 50.0%–67.2%), and 5-year local relapse-free survival rate was 75% (95% CI, 66.0%–83.1%). For local relapse-free survival we found five important factors independently related to worse prognosis: (1) clinically recurrent tumor (Fig. 2A); (2) high (Grade 2 or 3) histological grade; (3) surgical resection margins R1 (microscopically nonradical but macroscopically radical resection) (Fig. 2B); (4) unplanned excision without preoperative biopsy (Fig. 2C); and (5) myxoid/round cell or pleomorphic histologic subtype (Table 3). We identified one independent factor that negatively influenced disease-specific survival: AJCC Stage 3. For disease-free survival we identified three independent negative prognostic factors: (1) histological Grade 3; (2) presence of clinical local recurrence at the start of therapy in a sarcoma center; and (3) tumor skin infiltration. For local relapse-free survival, we found two independent factors with a negative impact: (1) presence of clinical local recurrence at the start of therapy in a sarcoma center; and (2) microscopically positive margin after surgery (Table 4).

**Table 4:** Multivariate analysis of prognostic factors for disease-specific survival (DSS), disease-free survival (DFS), and local relapse-free survival (LRFS)

Factor	Hazard ratio	95% CI		p value
DSS				
Primary tumor				
Clinical recurrence	1.124	0.540	2.340	0.760
Scar after nonradical surgery	0.160	0.021	1.229	0.078
AJCC stage				
2	2.117	0.706	6.345	0.180
3	5.772	2.075	16.057	0.001
DFS				
Grade				
2 versus 1	1.543	0.742	3.212	0.250
3 versus 1	3.808	1.925	7.531	0.000
Primary tumor				
Clinical recurrence	2.219	1.317	3.737	0.003
Scar after nonradical surgery	0.884	0.336	2.324	0.800
Skin infiltration				
Yes versus no	2.795	1.380	5.661	0.004
LRFS				
Primary tumor				
Clinical recurrence	2.835	1.389	5.786	0.004
Scar after nonradical surgery	1.436	0.458	4.503	0.530
R0/R1 margins				
R1 versus R0	2.090	1.014	4.307	0.046

R0 = microscopically radical resection; R1 = microscopically nonradical, but macroscopically radical resection; AJCC = American Joint Committee on Cancer.

We found differences (p = 0.01) for local relapse-free survival based on tumor status at the beginning of therapy in the tertiary center: the 5-year local relapse-free survival rate for a patient with a primary tumor treated in the sarcoma center was 86.1%, for those after resection of the scar after nonradical surgery at an outside sarcoma center it was 73.3%, and for clinical local relapse after primary surgery at an outside sarcoma center it was 52.1%. For disease-specific survival when calculated from the date of the first surgery we found no difference (p = 0.14) among the three groups: the 5-year disease-specific survival rate was 79.4 (95% CI, 70.5%–89.3%) for patients with a primary tumor treatment in our center, 100% for those after wide scar resection after unplanned nonradical surgery at an outside hospital, and for clinical local relapse after primary surgery at an outside sarcoma center 81.2% (95% CI, 70.7%–93.3%).

Seventy-three of the 181 patients (40%) had recurrences during followup, including 35 (19.3%) local recurrences. We found differences in first recurrence pattern according to liposarcoma pathological subtype; for the pleomorphic type the most common were distant metastases (mainly to lungs), myxoid/round cell type predominantly occurring in local recurrences, and among distant metastases lung metastases and abdominal wall/cavity metastases occurring with a similar frequency (Table 5).

**Table 5:** Localization of the first disease recurrences according to histological subtype of liposarcoma

Liposarcoma subtype (number)	Local relapse [number (%)]	Distant metastases				
		Lungs [number (%)]	Trunk wall [number (%)]	Abdominal cavity [number (%)]	Nodal metastases [number (%)]	Liver [number (%)]
Well differentiated (27)	4 (14.8)	0	0	0	0	0
Myxoid/round cell (104)	24 (23.1)	10 (9.6)	5 (4.8)	4 (3.8)	2 (2)	0
Pleomorphic (49)	7 (14.3)	13 (26.5)	1 (2)	1 (2)	1 (2)	2 (4)

## Discussion

Liposarcomas are a common histological type of soft tissue sarcomas, occurring mainly in adults and presenting a wide spectrum of clinical behavior. We have examined the outcomes of localized, resectable liposarcomas of the extremities or trunk wall treated with curative intent in a tertiary referral sarcoma center and asked (1) which prognostic factors influenced disease-specific survival, disease-free survival, and local relapse-free survival, including new AJCC staging system; (2) what was the impact of quality of surgery and tumor status (primary versus clinically recurrent versus scar after inadequate surgery); and (3) what is the type of disease recurrences according to pathological subtype of liposarcoma?

We recognize limitations of our study. First, although the study was retrospective, the patients were not preselected and long-term followup in our series exceeded the median time to recurrence, which in soft tissue sarcomas is usually not longer than 2 to 3 years. Overall and local relapse-free survivals at 5 years were 80% and 75%, respectively, which is similar or superior to other reported series [8, 15, 22, 23]. Second, although there are concerns about reliability of the AJCC staging system in sarcomas [14], we have confirmed the prognostic value of this current system. Third, tumor sites were limited to the extremity and trunk wall, what might have an impact on importance of some factors for patient prognosis, but it consequently created a more homogenous group for analysis. Fourth, myxoid and round cell were not analyzed separately, although round cell component has prognostic value, but for statistical reasons, these two subtypes were merged into one group, because the extent of myxoid/round cell component is generally considered a continuum of the same clinical/molecular entity; the percentage of these cells is somewhat arbitrarily recognized, especially on the basis of Tru-cut biopsy (which was used as preferential method for preoperative diagnosis), and tumor grade was included in multivariate analysis that interferes with myxoid/round cell proportions.

**Fig. 3:**
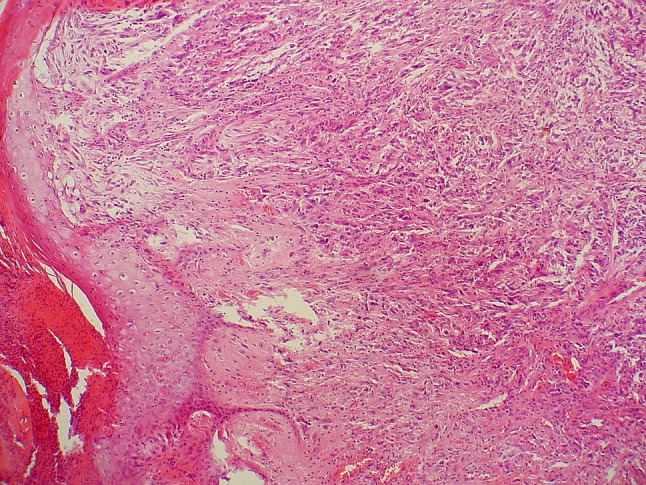
Micrograph demonstrating skin invasion by pleomorphic liposarcoma with ulceration (Stain, hematoxylin and eosin; original magnification, ×20).

Our data confirm the new AJCC staging system has strong prognostic value in terms of disease-specific survival for liposarcomas of the extremities/trunk wall. It was analyzed for the first time in literature regarding liposarcomas (Table 6). Beyond well-established prognostic factors such as tumor grade (the most reliable predictor of disease-free survival) and size [3, 25], we have also identified additional prognostic factors regarding pathological features, tumor status at presentation in the referral center, and quality of surgery, which can be ultimately incorporated into future revisions of staging system. A noteworthy finding is that sarcomatous skin infiltration (Fig. 3) is a sign of aggressive behavior with a detrimental effect on disease-free survival and it had also been indicated by Ruka et al. [29] as an independent prognostic factor.

**Table 6:** Main recent series of patients with liposarcomas

Series	Number of patients and followup time	Median age (years)	Site (%)	Subtypes	Primary/recurrent tumors	DSS/OS	LR	Prognostic factors for survival
Current study	181, median followup 52 months (minimum, 4 months)	58	Upper extremity (10%), lower extremity (76%), trunk (14%)	Well-differentiated, myxoid/round cell, pleomorphic	61% primary	DSS 5-year: 80%	19.3% Factors related to LRs: status at presentation, clinically recurrent tumor, high histological grade, microscopically positive surgical resection margin, unplanned excision without preoperative biopsy and nonwell-differentiated histological subtype	Negative factor for DSS (multivariate analysis): AJCC Stage 3
Moore Dala et al. [17]	801, median followup 45 months (all), 51 months (survivors); (minimum, 1 month)	56	All Upper extremity (7.9%), lower extremity (48.6%), retroperitoneum (33.5%), trunk (10.6%)	All	Primary only	DSS 5-year: 83%, 12-year: 72%	Not reported	Important factors for DSS (multivariate analysis): age, presentation status, primary site, histological variant, tumor burden, and gross margin status
Moreau et al. [18]	418, median followup 5.2 years (minimum, 0.1 year)	45	Upper extremity: 7%, lower extremity: 90%, trunk wall: 2%	Myxoid/round cell	112 (27%) after unplanned excision	DSS 5-year: 91% (pure myxoid) and 79% (round cell)	7.4%; positive microscopic margin strongly related to LRs; radiotherapy reduced LRs	Negative for DSS multivariate analysis): age at diagnosis > 45 years, tumor diameter > 10 cm, round cell percentage > 5%
Fiore et al. [9]	329, median followup 119 months (minimum, not available)	49	All Head/neck: 1%, trunk: 5%; retroperitoneum: 12%, extremities: 83%	Pleomorphic (P) and myxoid/round cell (M/R)	214 primary/ 115 recurrent	DSS 5-year: 83%; 10-year: 75%; Primary tumor: 5-year: 90%; recurrent tumors: 5-year: 72%	25%; multivariate negative factors for LR-free survival: recurrent tumor, non-extremity tumor site and lack of adjuvant radiotherapy	Negative for DSS (multivariate): recurrent tumors, size > 10 cm, positive surgical margins, higher tumor grade (if instead of histology)
Engström et al. [7]	319 (237 localized), median followup 8 years (survivors; minimum, 0 years)	54	Upper extremity: 8%, lower extremity: 84%, trunk: 8%	All	75% primary	OS/DSS 10-year: 64%/84%	13%; Negative factors for LR-free: surgery outside sarcoma center and histological type dedifferentiated liposarcoma; radiotherapy reduced LRs	Adverse factors for MFS: old age, large tumor size, high grade, nonwide surgical margin at reresection and histological type
Haniball et al. [12]	160, median followup 4.6 years (minimum, 2 years)	48.6	Upper extremity: 8%, lower extremity: 92%,	Myxoid/round cell	primary	DSS 5-year: 75%, 10-year: 56%	12%	Negative for DSS (multivariate analysis): presence of round cell component > 5%
Zagars et al. [33]	112, median followup 9.1 years (minimum, 2 years)	48	All (68% lower extremity)	All	83% primary/17% recurrent	OS 5-year: 79%, 10-year: 69%	13%; with LRs correlated pleomorphic histology, positive resection margins and prior disease recurrence	Negative for OS: age > 48 years, tumor size > 5 cm, pleomorphic histology
Nishida et al. [22]	53, mean followup 60 months (minimum, 12 months)	51	Upper extremity: 13.2%, lower extremity: 62.3%, trunk: 24.5%	Myxoid only	Not reported	OS 5-year: 90%, 10-year: 83%	13%	Negative for OS: age > 60 years
ten Heuvel et al. [31]	49, median followup 101 months (minimum, 4 months)	44	All (84% lower extremity)	Myxoid/round cell	Not reported	DSS: 5-year: 85%, 10-year: 72%	33%	Negative for DSS: older age at presentation, higher tumor grade and larger tumor size

DSS = disease-specific survival; OS = overall survival; LR = local recurrence; AJCC = American Joint Committee on Cancer; MFS = metastasis-free survival.

We confirmed an adverse impact of unplanned excision not preceded by appropriate biopsy and imaging for increasing the risk of local recurrences and that clinical local recurrence is the most important prognostic factor for subsequent local recurrence and disease-free survival [6, 8, 27, 28]. The tumor status (clinically recurrent versus primary) had also an impact on disease-specific survival in univariate and with borderline significance in multivariate analysis, which is similar to data from series of liposarcomas from the National Cancer Institute, Milan, Italy [9]. The presence of clinically recurrent tumors is likely one of the features of the aggressive behavior of liposarcoma and definitely warrants multimodal therapy. The detrimental effect of unplanned primary surgery is minimized by reexcision of the scar with adjuvant radiotherapy, and these patients do not have worse survival, which was also observed in some reports [8, 33]. However, the impact of the quality of surgery and status of the tumor at presentation in the sarcoma center on disease-specific survival is less clear when calculated from the date of initial (first) surgery; the obtained results were relatively good (5-year disease-specific survival approximately 80%) independent of the status at presentation in the sarcoma center [8]. We must take into account two possible biases leading to this result. First, patients referred after unplanned excision and undergoing reexcision of the scar tend to have smaller, anatomically favorably located or superficial tumors; this likely explains better disease-specific survival and of course this has an influence on lack of differences in disease-specific survival in the entire group of patients. The group of patients after unplanned excision is also biased as compared with primary tumors, because part of this group before referral may develop metastatic disease and can never be eligible for local therapy. Our data imply also the importance of negative margins of resection with curative intent. According to other series [15], it seems mostly important in reoperations for recurrent lesions.

Liposarcoma pathological subtype is an important factor in terms of prognosis and followup schedule; well-differentiated liposarcomas located on the extremities/trunk wall have the best prognosis, myxoid/round-cell liposarcomas intermediate, and the poorest prognosis was observed for pleomorphic liposarcomas. We have also confirmed that for myxoid/round cell liposarcoma, local recurrences are the most common type of relapse [22] and in case of recurrences, a high percentage of relapse was observed in unusual extrapulmonary sites [9, 24, 30, 31]. This is contrary to pleomorphic liposarcoma, which behaves as a typical high-grade sarcoma with a high propensity to lung metastases. These subtypes differ also in sensitivity to systemic chemotherapy (for instance, high response rates approaching 50% on trabectedin are observed in myxoid liposarcomas [11]) and radiotherapy [18, 26], which may influence their outcomes after recurrences. Myxoid/round-cell liposarcomas are the most common subtype of liposarcoma in the extremity/trunk wall localization, which shows histologically continuous morphologic spectrum-sharing characteristic chromosome rearrangement t(12;16) resulting in *DDIT3-FUS* (95%) or *EWS-CHOP* (5%) fusion [1, 10, 13] with diagnostic and possible treatment implications.

In conclusion, we have confirmed the value of the AJCC staging system for predicting disease-specific survival in patients with liposarcomas of the extremity or trunk wall. Wide reresection (plus radiotherapy) of the scar after nonradical primary tumor resection results in acceptable disease-free survival and local relapse-free survival in this type of soft tissue sarcoma. Patients with unplanned excision have a higher risk of local and distant recurrences but they can still be salvaged with a good final outcome when referred to a sarcoma unit and treated in an aggressive, combined way. We believe primary planned microscopically radical (R0) resection and multidisciplinary care in a tertiary referral center is crucial in the management of liposarcomas as well as all other soft tissue sarcomas.
